# Extracellular vesicle-associated miR-135b and -135a regulate stemness in Group 4 medulloblastoma cells by targeting angiomotin-like 2

**DOI:** 10.1186/s12935-020-01645-6

**Published:** 2020-11-20

**Authors:** Seung Ah Choi, Eun Jung Koh, Ryong Nam Kim, Jung Woo Byun, Ji Hoon Phi, Jeyul Yang, Kyu-Chang Wang, Ae Kyung Park, Do Won Hwang, Ji Yeoun Lee, Seung-Ki Kim

**Affiliations:** 1 Division of Pediatric Neurosurgery, Pediatric Clinical Neuroscience Center, Seoul National University Children’s Hospital, 101 Daehak-ro, Jongno-gu, Seoul, 03080 Republic of Korea; 2 Department of Neurosurgery, Seoul National University Hospital, Seoul National University College of Medicine, Seoul, Korea; 3 Regional Emergency Medical Center, Seoul National University Hospital, Seoul, Korea; 4 Department of Biomedical Engineering, Seoul National University, Seoul, Korea; 5 Department of Nuclear Medicine, Seoul National University College of Medicine, Seoul, Korea; 6 College of Pharmacy and Research Institute of Life and Pharmaceutical Sciences, Sunchon National University, Suncheon, Korea; 7 Department of Anatomy, Neural Development and Anomaly Lab, Seoul National University College of Medicine, Seoul, Korea

**Keywords:** Medulloblastoma, Brain tumour spheroid-forming cells, miR-135b, miR-135a, AMOTL2

## Abstract

**Background:**

Extracellular vesicles (EVs) secreted by tumours, including exosomes, are important factors that regulate cell–cell interactions in oncogenesis. Although EV studies are ongoing, the biological understanding of EV-miRNAs derived from brain tumour spheroid-forming cells (BTSCs) of medulloblastoma is poor.

**Purposes:**

We explored the specific cellular miRNAs and EV-miRNAs in medulloblastoma BTSCs to determine their potential biological function.

**Methods:**

Bulk tumor cells (BTCs) and BTSCs were cultured under different conditions from medulloblastoma tissues (N = 10).

**Results:**

Twenty-four miRNAs were simultaneously increased in both cells and EVs derived from BTSCs in comparison to BTCs. After inhibition of miR-135b or miR135a which were the most significantly increased in BTSCs, cell viability, self-renewal and stem cell marker expression decreased remarkably. Through integrated analysis of mRNAs and miRNAs data, we found that angiomotin-like 2 (AMOTL2), which was significantly decreased, was targeted by both miR-135b and miR-135a. STAT6 and GPX8 were targeted only by miR-135a. Importantly, low expression of AMOTL2 was significantly associated with overall poor survival in paediatric Group 3 and Group 4 medulloblastoma patients.

**Conclusion:**

Our results indicated that inhibition of miR-135b or miR-135a leads to suppress stemness of BTSC through modulation of AMOTL2.

## Background

Brain tumors contain small subpopulation cells with stem cell properties that play an important role in the tumor development, progression, metastasis and recurrence [1, 2]. These subpopulation cells have the ability spheroid-forming ability and are enrichment of cancer stem cells (CSCs), also called tumor initiating cells (TICs). They are known to be capable of self-renewal and differentiation into heterogeneous cancer cell lineages [3, 4]. Studies on these cells have been reported in medulloblastoma, the most prevalent malignant pediatric brain tumor [5, 6].

Advances in surgery and adjuvant therapies have led to a high cure rate. Recent 5-year survival of the patients with medulloblastoma is reaching 75%. However, the remaining 25% who do not respond to conventional therapies eventually deteriorate with recurrence and metastasis. Besides, the survivors are not free from severe adverse effects of chemotherapy and long-term sequelae of radiotherapy [7, 8]. Therefore, we need to better understand the molecular and cellular biology of the medulloblastoma for identifying the cause of treatment resistance and developing the safe and effective treatment modality.

The clinical complexity of many brain tumors is related to the peculiar microenvironment of the central nervous system (CNS) and to its intra/inter-cellular interactions; this contributes to make brain tumor a difficult medical challenge [9, 10]. Extracellular vesicles (EVs) are the one of forms of cell interaction that can provide an environment for cancer progression, metastasis and recurrence. Tumor-secreted EVs including exosomes play a pivotal role in the communication between tumor cells and stromal cells in local and distant microenvironments [11, 12]. The EVs contain selected miRNAs that could regulate biological functions such as cell proliferation, differentiation, apoptosis, metabolism and drug resistance [13, 14]. Previous studies have suggested that CSC-derived EVs act as a vehicle to deliver genetic information and produce a favorable microenvironment for cancer development and they were found to contain tumor cell specific miRNAs [15].

Extensive research of molecular genetics on medulloblastoma has been done and continue to progress, but studies on EVs of the medulloblastoma remains an area of further exploration. The major EV-miRNAs and their biological roles in medulloblastoma brain tumor spheroid-forming cells (BTSCs) have not yet been determined, and only exosome studies using a few established MB cell lines and a limited number of patient serum have been reported [10, 16]. This study was undertaken to gain insight into the identity of medulloblastoma-associated EV-miRNAs and to predict its applicability as a prognostic biomarker and therapeutic agent.

## Methods

### Patients and samples

Fresh tumour tissues (N = 10, Table 1) were obtained from medulloblastoma patients who underwent surgery at the Seoul National University Children’s Hospital. Eligible patients and/or their parents provided written informed consent to donate tumour tissue samples. The present study was approved by the Institutional Review Board (IRB) of the Seoul National University Hospital (IRB No. 1708-075-878). For primary cell culture, tumour tissues were processed within 6 h of resection. Snap-frozen tissues were used for subgroup analysis. Of the 10 patient samples, patient numbers 1–9 were used for all analyses, and number 10 was used only for functional analysis after validation of miR-135b, miR-135a and angiomotin-like 2 (AMOTL2) expression levels according to the analysis results.

**Table 1:** Patient information

Patient no	Gender	Age	Genetic profile	Histology	M stage^a^	Location	EOR	Follow-up
1	F	15 y	Medulloblastoma, WNT-activated	Classic	M0	Rt. Cbll	GTR	NED, alive (26 m)
2	M	8 y	Medulloblastoma, SHH-activated	Large cell/anaplastic	M0	Rt. Cbll	GTR	Recurred at 12 m, PD, expired (17 m)
3	F	5 y	Medulloblastoma, SHH-activated	Large cell/anaplastic	M0	Lt. Cbll	GTR	Recurred at 9 m, PD, expired (15 m)
4	M	5 y	Medulloblastoma, group 3	With extensive nodularity	M0	4 V	STR	NED, alive (48 m)
5	M	3 y	Medulloblastoma, group 3	Classic	M3	4 V	GTR	Recurred at 9 m, PD, expired (12 m)
6	M	10 y	Medulloblastoma, group 3	Large cell/anaplastic	M3	4 V	NTR	Recurred at 1 m, PD, alive (42 m)
7	F	12 y	Medulloblastoma, group 4	Large cell/anaplastic	M0	4 V	NTR	NED, alive (23 m)
8	F	7 y	Medulloblastoma, group 4	Classic	M0	4 V	NTR	NED, alive (26 m)
9	M	7 y	Medulloblastoma, group 4	With extensive nodularity	M1	4 V	NTR	Recurred at 22 m, PD, expired (37 m)
10	F	6 y	Medulloblastoma, group 4	Classic	M0	4 V	GTR	NED, alive (15 m)

*F* female, *M* male, *y* years, *MBEN* medulloblastoma with extensive nodularity, *Cbll* cerebellum, *4V* fourth ventricle, *EOR* extent of resection, *GTR* gross total resection, *STR* subtotal resection, *NTR* near total resection, *m* months, *NED* no evidence of disease, *PD* progressive disease

^a^ M stage (Chang’s classification) [47]. M0: no metastasis; M1: presence of tumor cells in the CSF; M2: nodular seeding in the cerebellar or cerebral subarachnoid space or in the third or lateral ventricle; M3: metastasis in spinal subarachnoid space; M4: metastases outside the cerebrospinal axis

### Cell cultures

Fresh tumour tissues (N = 10) were obtained and enzymatically dissociated to generate single cells. Single cells obtained from one patient were cultured separately into bulk tumour cells (BTCs) and brain tumour spheroid-forming cells (BTSCs) as previously described [17, 18]. BTCs were cultured in Dulbecco's modified Eagle's medium (Invitrogen) supplemented with 10% Exo-FBS™ Exosome-depleted foetal bovine serum (FBS; System Biosciences, Mountain View, CA). BTSCs were cultured as described previously [17, 18]. All cells were only used in early passages (< 4) for experiments and were maintained at 37 °C with 5% CO_2_ in a humidified atmosphere. In the additional functional study, only the BTSCs corresponding to Group 4 were used.

### Immunofluorescence

The cells were plated on 8-well Lab-Tek chamber slides (Nunc), and immunofluorescence studies were carried out using the following primary antibodies: Nestin (Chemicon; 1:200) and anti-Musashi (Neuromics, Bloomington, MN; 1:100) as described previously [17]. The secondary antibodies Alexa Fluor 488-conjugated goat anti-mouse IgG and Alexa Fluor 594-conjugated goat anti-rabbit (Invitrogen; 1:200) were applied, and then the cells were counterstained with an antifading solution containing 4-6-diamidino-2-phenylindole (DAPI; Vector Laboratories). The fluorescent images were obtained with a confocal microscope (Leica, Mannheim, Germany).

### Isolation of EVs, including exosomes

The EVs, including exosomes, were isolated from conditioned media using miRCURY exosome kits according to the manufacturer’s instructions (Exiqon, Vedbaek, Denmark). To remove cells and cellular fragments, samples were centrifuged at 3200*g* for 5 min. Precipitation buffer was added to the supernatants, and EVs were precipitated by cooling at − 20 °C for 12 h. EV pellets collected by centrifugation at 3200*g* for 30 min were dissolved in 20 µl of phosphate-buffered saline (PBS).

### Nanoparticle tracking analysis (NTA)

To analyse the size, concentration and distribution of EVs, NTA with a NanoSight NS3000 system (NanoSight Technology Malvern, UK) was employed. The samples were diluted to match 20–100 objects per frame and were gently injected into the NanoSight sample chamber. The detection threshold was maintained at 7 to ensure accurate and consistent detection of small particles, as previously described [19]. The data were analysed using the NTA analytical software (ver. 2.3).

### Transmission electron microscopy (TEM)

For morphology investigation, TEM (HT7700; Hitachi Ltd., Tokyo, Japan) was performed by the SNU imaging core facility. Briefly, the EVs were mixed with 4% paraformaldehyde and embedded for 20 min in a formvar–carbon-coated grid at room temperature. The embedded EVs were washed in PBS, fixed in 1% glutaraldehyde for 5 min, and stained with saturated aqueous uranyl oxalate. Samples were subsequently embedded in 0.4% uranyl acetate and 1.8% methylcellulose on ice for 10 min. Samples were dried at RT prior to visualization with TEM (JEOL, Peabody, MA). EVs were quantified by using the micro BCA protein assay (Thermo Fisher Scientific, Tokyo, Japan).

### Western blotting

Proteins from cells or EVs were extracted with RIPA buffer and equal amounts of proteins were used for western blot analysis. Primary antibodies were used against Flotillin-1 (1:500, Abcam, Cambridge, MA), Calnexin (1:500, Cell Signaling Technology, Danvers, MA) and β-actin (1:10,000, Sigma-Aldrich, St. Louis, MO). The membranes were incubated with a secondary antibody conjugated with horseradish peroxidase, and visualized using the enhanced Novex™ ECL Chemiluminescent Substrate Reagent Kit (Invitrogen).

### NanoView analysis

EVs were detected using the ExoView chip (NanoView Biosciences, Brighton, MA) according to the manufacturer's protocol and as previously described [20, 21]. Thirty-five microliters of diluted samples (1:2 dilution) were incubated overnight on microarray chips (NanoView) coated with capture antibodies for Alix-PE (Green, Biorbyt, St Louis, MO), Syntenin-CF555 (Green, NanoView), CD63-AF647 (Red, NanoView), CD9-AF488 (Blue, NanoView), or negative control IgG1 (NanoView). Once affinity captured, EVs were fixed and permeabilized with the ExoView Cargo Kit to enable access of antibodies to the EVs cargo. Chips were prescanned for background signal and then the conditioned media in incubation solution was dropped onto the chip surface and incubated overnight. The chips were washed, followed by antibody incubation in IF blocking solution. The chips were washed again followed by a rinse in filtered DI water and then dried. The number of positive particles detected for each fluorescence channel and quantified using the ExoView R100 reader using nScan 2.8.4 acquisition software (NanoView). The data were then analysed using NanoViewer 2.8.11 with sizing thresholds set to 50 to 200 nm diameter.

### RNA isolation

Total RNA, including miRNAs, was isolated using a miRNeasy kit (Qiagen, Hilden, Germany) according to the manufacturer’s instructions. The quality of the purified RNA was confirmed by a Nanodrop 2000 Spectrophotometer and an Agilent 2100 Bioanalyzer (Agilent Technologies, Santa Clara, CA).

### NanoString nCounter analysis

The miRNA profiling analysis was performed by the NanoString nCounter (NanoString Technologies, Inc., Seattle, WA) using 800 human v3 miRNA expression assays according to the manufacturer's protocol. In brief, the NanoString nCounter platform involved mixing total RNA with pairs of capture and reporter probes tailored to each miRNA; this was followed by hybridizing, washing away excess probes, immobilizing probe-bound miRNAs on a surface, and scanning the colour-coded bar tags of the reporter probes. A total of 80–100 ng of total RNA per sample from snap-frozen tissue was used as input material, and 3 µl of total volume was used for each sample. All hybridization reactions were incubated at 65 °C for 18 h, and a max-density scan (555 fields of view) was selected [22].

To identify the molecular subgroups for medulloblastoma, nCounter Elements TagSets was applied as previously described [23]. The algorithm for class prediction analysis was provided by Dr. M. Taylor (Toronto, Canada) and was used for analysis.

### mRNA profiling with RNA sequencing analysis

Total RNA from the BTSCs and BTCs was used for the preparation of paired-end libraries with the TruSeq Stranded mRNA LT Sample Prep Kit. In detail, purified RNAs were randomly fragmented, and the fragmented RNAs were transformed into cDNAs by reverse transcription. The cDNA fragments’ both ends were ligated with adaptors. After PCR amplification for a quantity suitable for sequencing, cDNA fragments with insert sizes of 200–400 bp were selected and sequenced in a paired-end manner on a NovaSeq 6000 System.

After trimming the adaptor and removing the low-quality sequences from the raw data, the trimmed RNAseq data were mapped to the human genome (hg19) by using Bowtie2 and HISAT2 software. Using StringTie software, we performed transcript assembly and calculated Fragments Per Kilobase of transcript per Million mapped reads (FPKM) values.

### Analysis of differentially expressed mRNAs and miRNAs

Using the limma package written in R programming language (version 3.5.1), we identified differentially expressed genes in the normalized mRNA and miRNA datasets. The mRNAs and miRNAs with a log2-fold change  ≥ 2 or ≤ − 2 and a False Discovery Rate (FDR) < 0.05 were considered differentially expressed.

### Bioinformatic analysis used to predict target genes of miRNAs

Using 12 representative miRNA target databases, PhenomiR, DIANA-microT, ElMMo, MicroCosm/miRBase, miRanda, miRDB, PicTar, PITA, TargetScan, miRecords, and miRTarBase, we identified candidate target genes for the differentially expressed miRNAs. To avoid biased prejudice or insufficiency in analysing target genes, we have taken advantage of the comprehensive resources of the above 12 miRNA target databases rather than relying upon one or two individual databases. In choosing the candidate target genes, we maintained the following criteria: either target gene predicted simultaneously by at least 3 of the abovementioned representative miRNA target databases or target gene verified by a research validation in at least one previous investigation.

### Integration analysis of miRNAs and mRNAs

By comparing the abovementioned candidate miRNA target genes and the mRNAs (with log2-fold change, ≥ 2 or ≤ − 2, P value < 0.05 and False Discovery Rate [FDR] < 0.05) in the RNAseq dataset for our samples, we finally selected target genes. To guarantee that we pursued high-quality target genes, we have given a priority (for validation test) to the target genes showing highly statistically significant negative correlation (Pearson correlation coefficient < − 0.7) in expression between miRNAs and mRNAs in our samples. Using the Database for Annotation, Visualization and Integrated Discovery (DAVID) and Kyoto Encyclopedia of Genes and Genomes (KEGG) databases, we performed gene ontology (GO) and pathway analyses for the target genes.

### Reverse transcription and quantitative real-time polymerase chain reaction (qRT-PCR)

Total RNA was extracted, and cDNA was generated using an RT kit (Qiagen) according to the manufacturer’s instructions. qRT-PCR was performed to measure selected gene expression by using the ABI real-time PCR detection system and the SYBR Green master mix kit with specific primers. RUN6B was used to normalize cellular miRNA expression. Since there are no known control or housekeeping miRNAs in EVs, we adopted the strategy of using *C. elegans* miRNAs directly spiked into the Qiazol prior to RNA extraction to be normalizing controls, as previously described [24]. The relative expression of each gene was calculated using the comparative threshold cycle method, as previously described.

### In vitro transfection of miRNA inhibitors

For miRNA inhibitor transfection, BTSCs were grown to 50% confluency, and then 20 nM of anti-miR-135b and anti-miR-135a (Qiagen) were transfected into BTSCs to inhibit their respective miRNAs using the RNAiMAX reagent (ThermoFisher, Waltham, MA) according to the manufacturer’s protocol. miScript inhibitor negative control (anti-miR-NC) under the same concentrations and conditions was used as a control. The transfection efficiency was evaluated by qRT-PCR as described above, and all functional studies were performed at 48 h.

### Cell viability assay

The cell viability was assessed after miRNA inhibitor transfection using an EZ-cytox kit (Daeil Lab Service) according to the manufacturer’s protocol (All experiments were conducted in triplicate. Cell viability was calculated as the ratio of the absorbance from the miRNA inhibitor-treated cells to the absorbance of the control group cells.

### Cell apoptosis assay

Apoptosis was analyzed by the annexin V-FITC/propidium iodide (PI) apoptosis kit (BD Biosciences, San Jose, CA) according to the manufacturer’s instructions and as previously described [18]. In brief, the cells were transfected with anti-miRNA, anti-miR-135b or anti-miR-135a for 48 h and then the stained cells were detected by FACSCanto (BD), and flow cytometric plots were generated by FlowJo software.

### Cell senescence assay

Senescence-associated β-galactosidase (SA-β-gal) activity was detected using the cellular senescence assay kit (Chemicon, Temecula, CA), according to the manufacturer’s instructions and as previously described [25]. In brief, the cells were transfected with anti-miRNA, anti-miR-135b or anti-miR-135a for 48 h and then seeded in seeded in 6-well plates, and incubated for 16 h at 37 °C. Representative microscopic fields were photographed.

### Limiting dilution assay

To assess the self-renewal ability, limiting dilution assays were performed as previously described [17]. After miRNA inhibitor transfection, the cells (1 × 10^3^) were cultured onto 48-well ultra-low cluster plates (Costar). Data were analysed using the extreme limiting dilution software web interface (https://bioinf.wehi.edu.au/software/elda/).

### Survival analysis and AMOTL2 gene expression association in two paediatric medulloblastoma cohorts

Log-rank tests were performed to examine the association between AMOTL2 expression level and overall survival outcome in two independent paediatric medulloblastoma cohorts from previous studies, including 30 Korean patients (Seoul National University Children’s Hospital [SNUCH] cohort) [26] and 530 patients (age < 18) from Medulloblastoma Advanced Genomic International Consortium (MAGIC) [27, 28]. In the log-rank tests, the patients were divided into high, medium and low AMOTL2 expression groups at one of three cut-off points, 0.25, 0.5, or 0.75 quantile, which were based on the samples in each cohort. Subsequently, survival distributions were compared between the two groups, and the cut-off point with the most significant P-value was selected for each final log-rank test. All analyses were repeated for each medulloblastoma subgroup. Subgrouping of the 30 Korean medulloblastoma cohorts was performed using unsupervised hierarchical clustering and partitioning around medoid (PAM) methods and was based on the expression profiles of 22 marker genes from a CodeSet [29].

### Statistical analysis

All statistical analyses were performed with SPSS 19.0 software (SPSS, Chicago, IL) and GraphPad Prism 5.0 (GraphPad Software, Inc., San Diego, CA). Data are presented as the mean ± SD. One-way analysis of variance followed by a Tukey post hoc test was used to determine the differences occurring between more than two groups, and a t-test was used to determine the difference between two groups. All experiments were repeated at least three independent times. P-values < 0.05 were considered statistically significant.

## Results

### Characterization of primary cultured cells and EVs from medulloblastoma patients

**Fig. 1:**
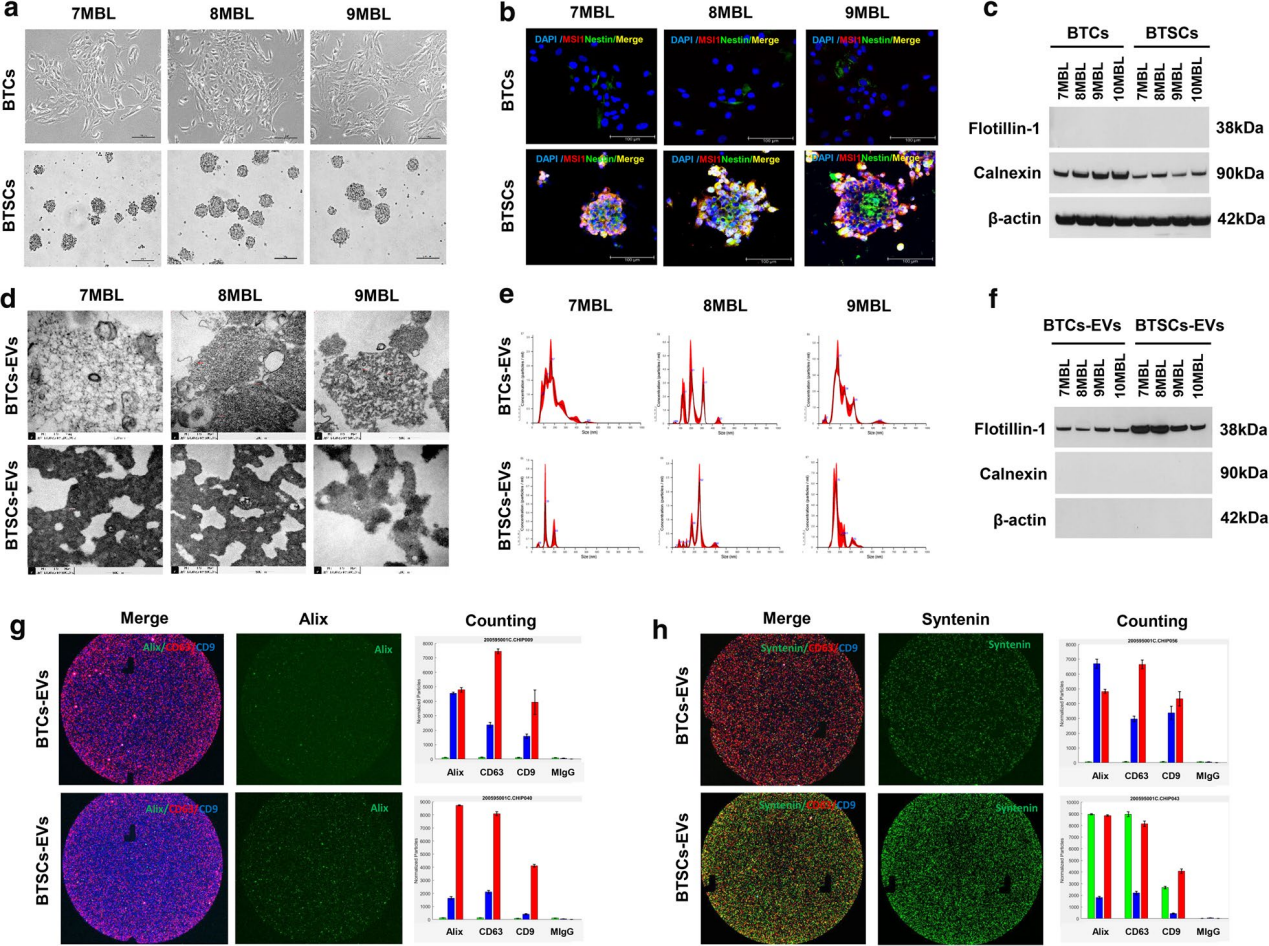
Characterization of bulk tumour cells (BTCs), brain tumour spheroid-forming cells (BTSCs) and extracellular vesicles (EVs) released from BTCs or BTSCs (BTCs-EVs or BTSCs-EVs, respectively). The characteristics of BTCs and BTSCs (**a**–**c**) are compared, and the characteristics of each EV (**d**, **e**) derived from them are analysed. **a** Representative microscopy images show the phenotype of adherent BTCs and spheroid-forming BTSCs. Scale bars: 100 μm. **b** The cells expressing the stem cell marker nestin (green) are very rare, and musashi (MSI1, red) expression is scarcely present in adherent BTCs, whereas both nestin and MSI1 are strongly expressed in BTSCs. Cells were counterstained with DAPI (blue). Scale bars: 100 μm. **c**, **f** Western blot analysis shows that calnexin and β-actin are detected, but flotillin-1 is not found in cell lysates (BTCs and BTSCs). **d** EVs appear as isolated vesicles with characteristic round-shaped structures of exosomes in a transmission electron microscopy (TEM) image. **e** Nanoparticle tracking analysis (NTA) shows that the size distribution ranges from 10 to 200 nm in diameter. **f** Exosomal marker protein flotillin-1 is observed, but calnexin and β-actin are not observed in EVs (BTCs-EVs and BTSCs-EVs), indicating the presence of exosomes in EVs. **g**, **h** NanoView analysis system detect EVs captured by the CD63 antibody and observed simultaneously by fluorescently tagged Alix-PE (green), CD63-AF647 (red), and CD9-AF488 (blue). Representative images provide co-localization information for **g** Alix(Green)/CD63(Red)/CD9(Blue) and **h** Syntenin(Green)/CD63(Red)/CD9(Blue). The normalized number of particles is shown in the bar graphs

Prior to all experiments, we performed a characterization analysis of isolated cells and EVs derived from cells, including exosomes. The BTCs and BTSCs cultured under different culture conditions are attached adherent cells and floating spheroid-forming cells, respectively, at passage number 5 (Fig. 1a). The floating BTSCs are considered to be enriched with stem/progenitor cancer cells [17]. We identified the expression of stem cell markers such as nestin and musashi between BTCs and BTSCs. Nestin was expressed in a very small number of cells of BTCs, and no musashi expression was found. On the other hand, BTSCs had very strong expression of both nestin and musashi (Fig. 1b). Immunoblotting showed calnexin expression but no flotillin-1 expression in both BTCs and BTSCs (Fig. 1c).

The characterization of EVs derived from BTCs and BTSCs was confirmed. TEM analysis exhibited round, double-lipid membrane vesicles (Fig. 1d). NTA showed a particle size distribution ranging from 10 to 200 nm in diameter, with most particles detected in the size range corresponding to exosome dimensions (85.8 ± 6.8 nm; Fig. 1e). Immunoblotting revealed the vesicular enrichment of flotillin-1 and the absence of calnexin and β-actin (Fig. 1f). In addition, ExoView assay provided the presence of internal proteins (Alix and Syntenin) at the EVs level and co-localization with tetraspanins (CD63 and CD9). Immunocaptured vesicles of Alix/CD63/CD9 (Fig. 1g) and Syntenin/CD63/CD9 (Fig. 1h) were observed in both EVs secreted from BTCs and BTICs.

### Comparative analysis of cellular miRNAs and EV-miRNAs between BTCs and BTSCs

**Fig. 2:**
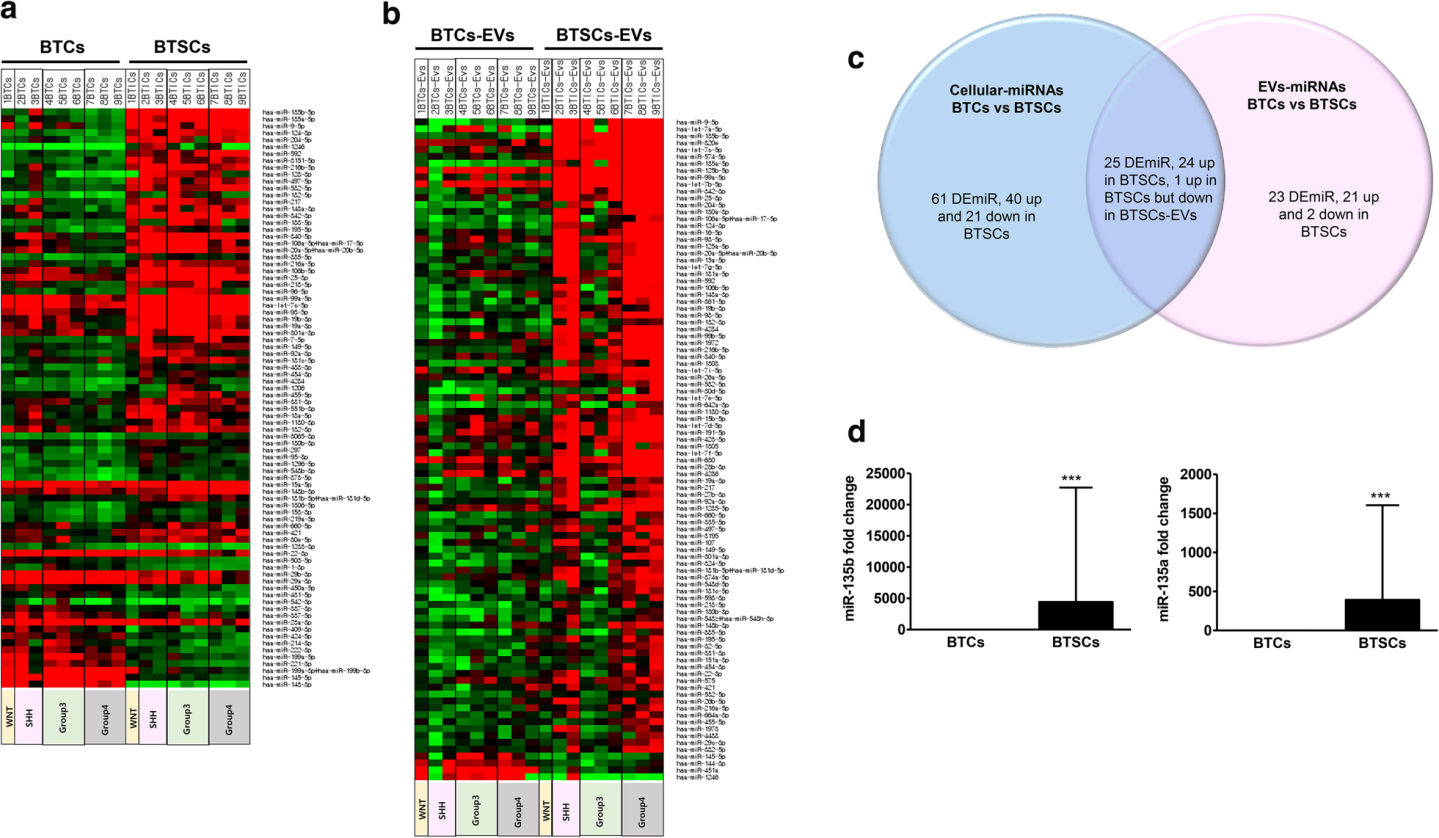
miRNA expression signature of bulk tumour cells (BTCs) and brain tumour spheroid-forming cells (BTSCs) from medulloblastoma patients. Heatmap of miRNA analysis represents the significantly different expression levels of miRNAs in **a** BTSCs compared with BTCs and **b** BTSCs-extracellular vesicles (EVs) compared with BTCs-EVs. The colour values display log2 (normalized data). The red or green colour indicates relatively high or low expression, respectively (differences significant with P < 0.05). **c** Venn diagram showing the unique and overlap of miRNAs between BTSCs (blue) and BTSCs-EVs (pink). **d** Confirmation of the expression level of miR-135b and miR135a performed by RT-qPCR shows significantly different expression levels in BTSCs compared with BTCs. ***P < 0.001

First, we performed gene profiling on 22 subgroup-specific signature genes to identify medulloblastoma subgroups. We confirmed that there were 1 WNT, 2 SHH, 3 Group 3 and 4 Group 4 medulloblastoma through class prediction analysis (Table 1). Next, we performed miRNA profiling of cells and EVs utilizing NanoString nCounter miRNA arrays in individuals with BTSCs compared to those with BTCs. Differentially expressed miRNA (DEmiR) analysis showed that 86 miRNAs were differentially expressed with statistical significance in BTSCs compared to BTCs (Fig. 2a, b). In the case of the EVs, 48 miRNAs were differentially expressed in BTSC-derived EVs (BTSCs-EVs) compared to in BTC-derived EVs (BTCs-EVs). In both BTSCs-EVs and BTSCs, the expression of 25 miRNAs was found to be highly significantly different, with an interesting observation that 24 of these miRNAs were upregulated compared to in BTCs (Fig. 2c and Table 2). Of these, miR-1246 was upregulated in the BTSCs but downregulated in the BTSCs-EVs. For further functional analysis, we focused on miR-135b and miR-135a, which showed the highest statistical significance and fold change in both BTSCs and BTSCs-EVs. The differential expression of the two miRNAs in BTCs and BTSCs was verified by RT-qPCR (Fig. 2d, P < 0.001).

**Table 2:** Unique and overlapping miRNAs in brain tumor spheroid-forming cells (BTSCs) compared to bulk tumor cells (BTCs)

Extracellular vesicle (EVs) only		Both		Cell only	
Up-regulated	Down-regulated	Up-regulated	Down-regulated	Up-regulated	Down-regulated
hsa-let-7a-5p	hsa-miR-144-3p	hsa-miR-135b-5p	hsa-miR-1246 (up in cells, down in EVs)	hsa-miR-3151-5p	hsa-miR-1233-3p
hsa-miR-320e	hsa-miR-451a	hsa-miR-135a-5p		hsa-miR-128-3p	hsa-miR-22-3p
hsa-miR-574-5p		hsa-miR-9-5p		hsa-miR-497-5p	hsa-miR-503-5p
hsa-miR-125b-5p		hsa-miR-124-3p		hsa-miR-182-5p	hsa-miR-1-3p
hsa-let-7b-5p		hsa-miR-204-5p		hsa-miR-217	hsa-miR-29b-3p
hsa-miR-130a-3p		hsa-miR-592		hsa-miR-183-5p	hsa-miR-29a-3p
hsa-miR-125a-5p		hsa-miR-216b-5p		hsa-miR-195-5p	hsa-miR-450a-5p
hsa-let-7g-5p		hsa-miR-582-5p		hsa-miR-885-5p	hsa-miR-431-5p
hsa-miR-181a-5p		hsa-miR-148a-3p		hsa-miR-216a-5p	hsa-miR-542-3p
hsa-miR-361-5p		hsa-miR-342-3p		hsa-miR-218-5p	hsa-miR-337-3p
hsa-miR-98-5p		hsa-miR-340-5p		hsa-miR-96-5p	hsa-miR-337-5p
hsa-miR-99b-5p		hsa-miR-106a-5p+hsa-miR-17-5p		hsa-miR-19a-3p	hsa-miR-23a-3p
hsa-miR-1972		hsa-miR-20a-5p+hsa-miR-20b-5p		hsa-miR-301a-3p	hsa-miR-409-3p
hsa-miR-1303		hsa-miR-106b-5p		hsa-miR-7-5p	hsa-miR-424-5p
hsa-let-7i-5p		hsa-miR-25-3p		hsa-miR-149-5p	hsa-miR-214-3p
hsa-miR-26a-5p		hsa-miR-99a-5p		hsa-miR-92a-3p	hsa-miR-222-3p
hsa-miR-30d-5p		hsa-let-7c-5p		hsa-miR-181c-5p	hsa-miR-199a-5p
hsa-let-7e-5p		hsa-miR-93-5p		hsa-miR-488-3p	hsa-miR-221-3p
hsa-miR-642a-3p		hsa-miR-19b-3p		hsa-miR-454-3p	hsa-miR-199a-3p+hsa-miR-199b-3p
hsa-miR-15b-5p		hsa-miR-4284		hsa-miR-1206	hsa-miR-145-5p
hsa-let-7d-5p		hsa-miR-1180-3p		hsa-miR-455-5p	hsa-miR-143-3p
		hsa-miR-132-3p		hsa-miR-331-3p	
		hsa-miR-16-5p		hsa-miR-551b-3p	
		hsa-miR-15a-5p		hsa-miR-18a-5p	
				hsa-miR-455-3p	
				hsa-miR-3065-3p	
				hsa-miR-130b-3p	
				hsa-miR-297	
				hsa-miR-95-3p	
				hsa-miR-1296-5p	
				hsa-miR-548b-3p	
				hsa-miR-873-5p	
				hsa-miR-148b-3p	
				hsa-miR-181b-5p+hsa-miR-181d-5p	
				hsa-miR-1306-5p	
				hsa-miR-153-3p	
				hsa-miR-219a-5p	
				hsa-miR-660-5p	
				hsa-miR-421	
				hsa-miR-30e-5p	

*EVs* extracellular vesicles

### Functional regulation of BTSCs by miR-135b or miRNA-135a inhibition

**Fig. 3:**
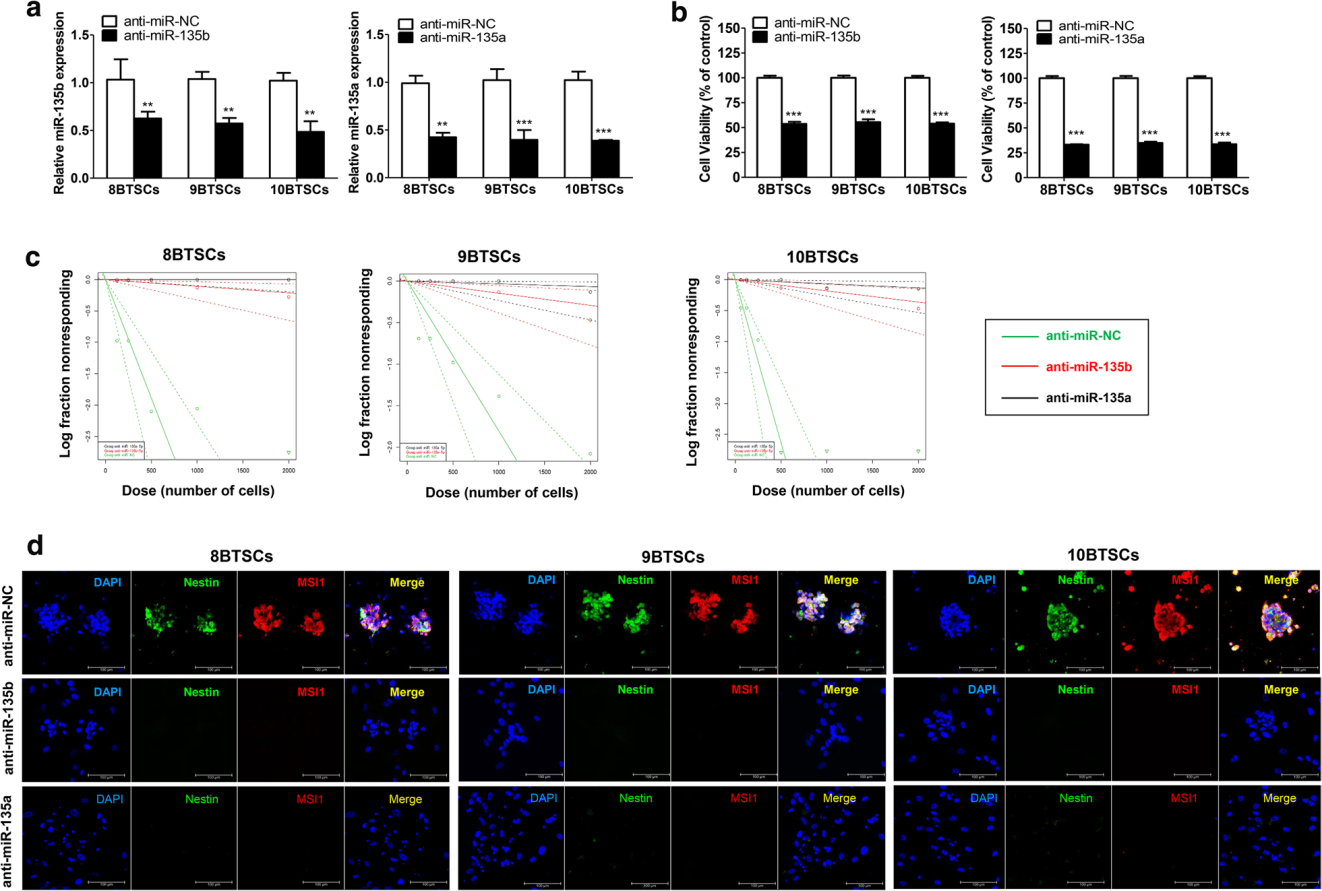
Functional analysis of miR-135b and miR-135a in brain tumour spheroid-forming cells (BTSCs). **a** RT-qPCR shows significantly decreased miR-135b or miRNA-135a levels in BTSCs transfected with each anti-miRNA compared with the anti-miR-negative control (NC). **b** Inhibition of miR-135b or miR-135a leads to a significant reduction in cell viability in comparison with controls. **c** Extreme limiting dilution assay assessment shows significantly reduced tumour sphere formation ability after miR-135b or miRNA-135a inhibition. **d** Immunofluorescence analysis reveals that the ability of sphere formation is decreased, and the expression of nestin and musashi (MSI1) is markedly reduced by inhibition of miR-135b or miR-135a. Scale bars: 100 μm. **P < 0.01. ***P < 0.001

We next investigated whether secreted miR-135b and miR-135a function at the microenvironment level by possibly impacting the stemness of BTSCs. To determine the effect of miR-135b and miR-135a on the function, mechanism and signal pathway of BTSCs, we conducted in vitro inhibition experiments on Group 4 BTSCs. After anti-miR (miRNA inhibitor) treatment, miR-135b and miR-135a expression levels significantly decreased compared to anti-miR-NC expression levels (Fig. 3a, P < 0.01, P < 0.001). Cell viability analysis showed that the cell survival rate was significantly reduced by the inhibition of miR-135b or miR-135a at 48 h in all BTSCs (Fig. 3b, P < 0.001). Subsequently, apoptosis and senescence were examined. The early apoptotic rate increased 2.4–5.4 times with anti-miR-135b treatment and 2.5–4.7 times with anti-miR-135a treatment compared with that of the anti-miRNA-NC treatment (Additional file 1: Fig. S1A). However, no differences were observed in senescence (Additional file 1: Fig. S1B). Upon treatment, the sphere-forming ability decreased (Fig. 3c), and the expression of nestin and musashi decreased in all of the examined BTSCs (Fig. 3d). Overall, these results suggest that the inhibition of miR-135b or miR-135a can suppress the self-renewal capacity and expression of stem cell-related markers of BTSCs.

### Integrative analysis of miRNA and mRNA expression with predicted miRNA targets

**Fig. 4:**
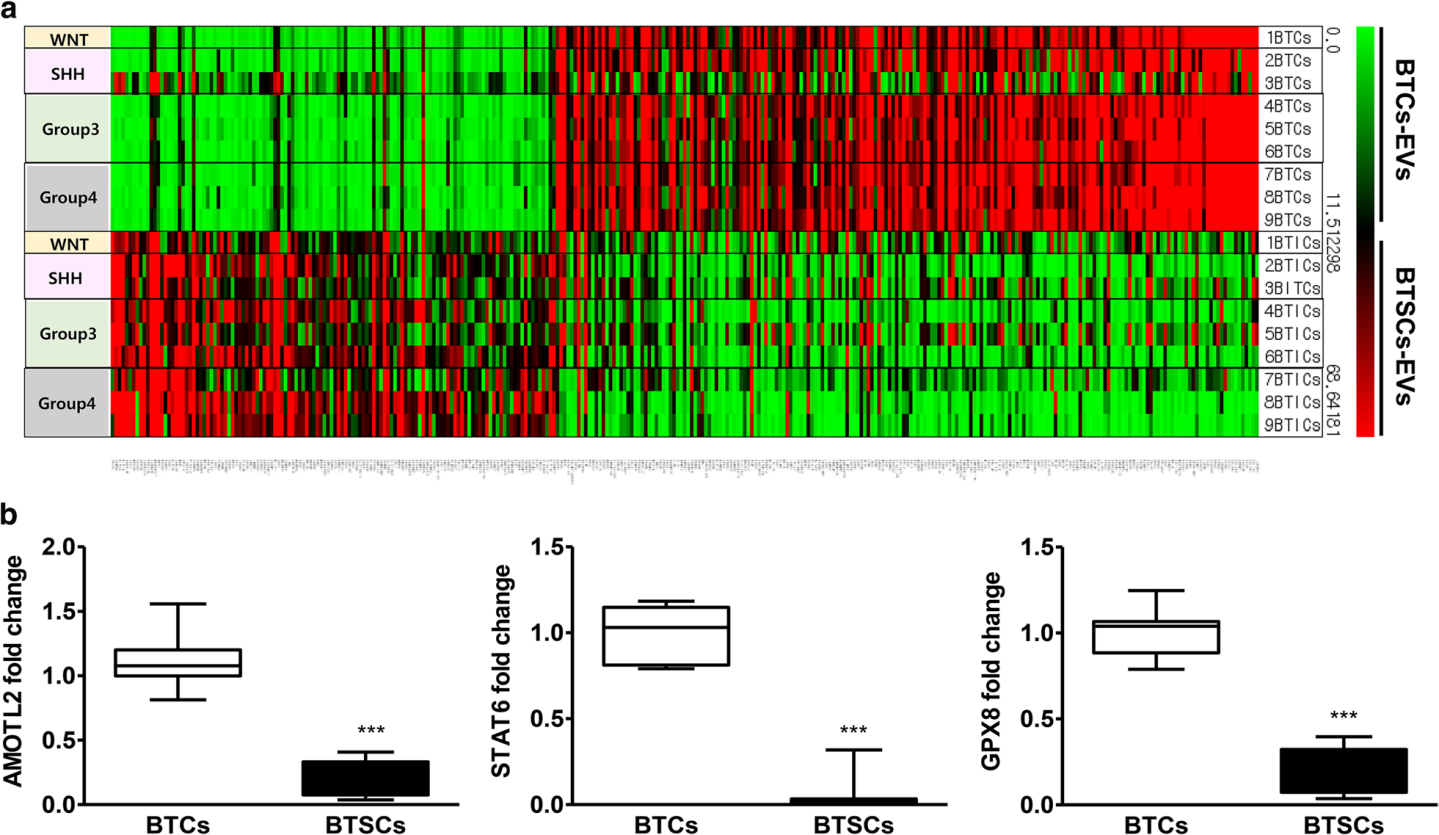
Integrated analysis and validation of mRNA-targeted miRNAs in brain tumour spheroid-forming cells (BTSCs). **a** Differential gene expression between bulk tumour cells (BTCs) and BTSCs was determined by RNA-seq. According to the integrated analysis, there are 199 genes that are predicted to be targets of the 23 upregulated miRNAs. The number of previously identified targets for miR-135b and miR-135a targets is 1 and 3, respectively. **b** The predicted target genes for miR-135b or miR-135a were identified as AMOTL2, STAT6, GPX8, and their expression was verified by RT-qPCR. Compared with BTCs, the expression of AMOTL2, STAT6, and GPX8 was observed to be significantly reduced in BTSCs. ***P < 0.001

**Fig. 5:**
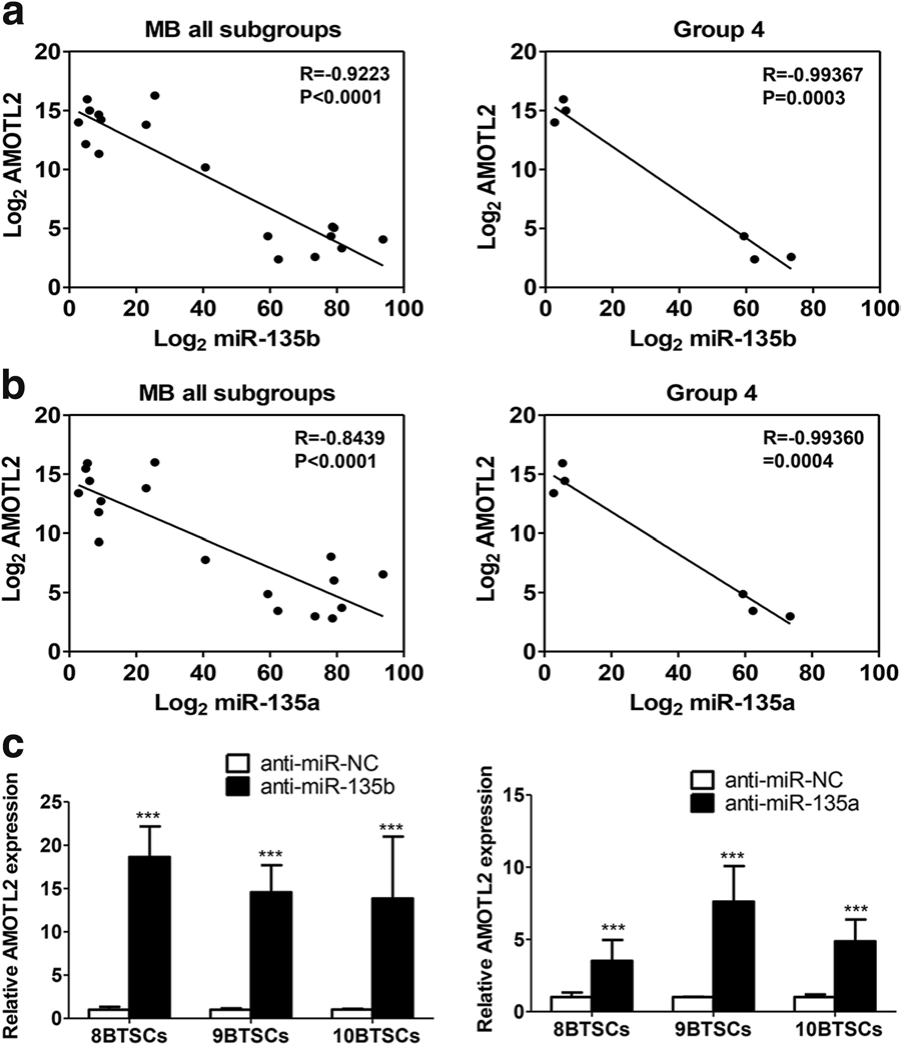
The correlation coefficients between target genes AMOTL2 and miR-135b and miR-135a in medulloblastoma all subgroups and Group 4. **a** The R value of AMOTL2, the target of both miR-135b and miR-135a, is − 0.92006 (P < 0.0001) for miR-135b and − 0.82885 (P < 0.0001) for miR-135a. The R values are also shown for each subgroup of medulloblastoma. **b** RT-qPCR reveal increased expression of AMOTL2 in BTSCs after miR-135b or miR-135a inhibition. ***P < 0.001

We analysed the predicted target mRNAs of the upregulated miRNAs with downregulated mRNA expression from the microarray and vice versa [30–33]. Based on the above method, a total of 325 significant miRNA–mRNA pairs were predicted, consisting of 109 differentially expressed miRNAs and 1640 mRNAs. Among the 750 significantly decreased mRNAs, 199 genes were predicted to be targets of 23 upregulated miRNAs (Fig. 4a). The target mRNA of miR-135b and/or miR-135a was selected, verified and analysed (N = 10, Fig. 4b, P < 0.001). We found that miR-135b targets only one gene, AMOTL2, and miR-135a targets AMOTL2, signal transducer and activator of transcription 6 (STAT6) and glutathione peroxidase 8 (GPX8). Notably, AMOTL2 was targeted by both miR-135b and miR-135a. AMOTL2 expression levels were negatively correlated with those of miR-135b (R = − 0.92006; P < 0.0001) and miR-135a (R = − 0.82885; P < 0.0001) (Fig. 5a, b, Additional file 2: Fig. S2A and S2B). STAT6 (R = − 0.85386; P < 0.0001) and GPX8 (R = − 0.81841; P < 0.0001) were also associated with miR-135a (Fig. 5a, b and Additional file 2: Fig. S2C). Although the number of samples was small, there were no significant differences according to subgroups, and their association’s patterns were found to be similar in all groups. These results implied that high expression levels of miR-135b and miR-135a target and downregulate the expression of AMOTL2. To further determine whether AMOTL2 is regulated by miR-135b or miR-135a, the expression level of AMOTL2 after inhibition of miR-135b and miR-135a was investigated by RT-qPCR. The expression of AMOTL2 was 15.7- and 5.3-fold higher following inhibition of miR-135b and miR-135a, respectively, when compared with the control (Fig. 5c). These results show that miR-135a and miR-135a function in BTSCs, at least partially through down-regulating the expression of AMOTL2.

To explore the biological pathway associated with miR-135b-targeted AMOTL2, we used KEGG pathway analysis. As a result, we found the Hippo and tight junction pathways involved in AMOTL2. Based on our RNA-seq results, we identified upregulated (red) and downregulated (green) genes (Additional file 3: Fig. S3).

### Association of AMOTL2 expression with overall survival outcome

**Fig. 6:**
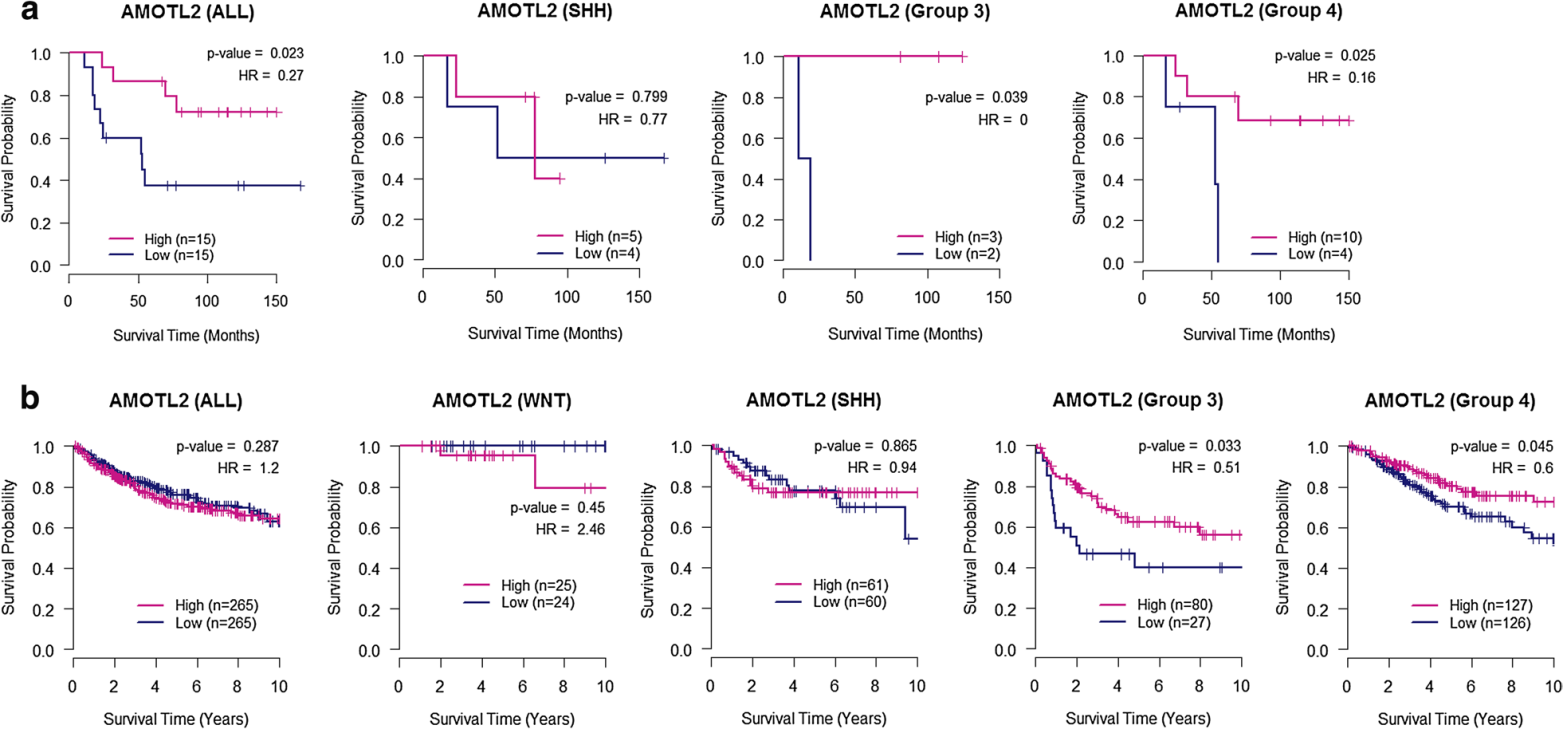
Association analysis between the expression level of AMOTL2 and the overall survival of paediatric medulloblastoma patients in two independent cohorts. **a** Kaplan–Meier plots with log-rank tests in two groups of 30 Korean patients with high or low expression of AMOTL2 (Seoul National University Children’s Hospital [SNUCH] cohort). **b** Kaplan–Meier plots with log-rank tests in two groups of patients with high or low expression of AMOTL2 in the Medulloblastoma Advanced Genomics International Consortium (MAGIC) cohort

We evaluated the association between the expression level of AMOTL2 and clinical outcome in two independent cohorts (SNUCH and MAGIC) [26–28]. Survival analysis revealed that high expression of AMOTL2 was significantly associated with a favourable prognosis in paediatric medulloblastoma patients in Group 3 and Group 4, which was consistently observed in the two independent cohorts (Fig. 6). Unlike Group 3 and Group 4, high expression of AMOTL2 was associated with poor prognosis in WNT, and there was no significant correlation in SHH group.

## Discussion

In this study, we profiled both cellular miRNAs and EV-miRNAs from BTCs and BTSCs and found that miR-135b and miR-135a were highly expressed in the BTSCs and BTSCs-EVs. Importantly, inhibition of miR-135b and miR-135a reduces the stemness of medulloblastoma BTSCs. Furthermore, an integrated analysis suggested that miR-135b and miR-135a regulate AMOTL2. Our results might be the first evidence to demonstrate that the secretion of these miRNAs through EVs could potentially be a consequence of BTSCs attempting to maintain their stemness or signalling to other cells.

Previous evidence has indicated that miR-135b is highly expressed in the CSCs of paediatric solid tumours [34, 35]. The direct inhibition of miR-135b may itself serve as a therapeutic intervention against a network of events driving oncogenesis and may even target self-renewing CSCs [35]. In the case of miR-135a, there has been controversy over whether onco-miRs or tumour suppressor-miRs are present in several other tumours. miR-135a is known as onco-miR in breast cancer [36] and colorectal carcinomas [37], but it is known as tumour suppressor in renal cell carcinoma [38] and gastric cancer [39]. The opposite function of miR-135a was identified in different genetic contexts in a study of glioblastoma [40]. This controversy may also be due to the difference between the analysed sample and the comparison group.

Based on the results of our analysis, we conducted functional verification focusing on miR-135b and miR-135a by focusing on the highest log fold-change and the p-value. Our stemness assay confirmed that in vitro blockade of miR-135b or miR-135a demonstrated a markedly diminished ability of the medulloblastoma cells to form spheres, thereby alluding to the role of miR-135b and miR-135a in self-renewal and proliferation.

AMOTL2 belongs to the angiomotin (AMOT) family of membrane-associated scaffold proteins. AMOTL2 is commonly accepted as a tumour suppressor [41, 42]. AMOTL2 has been shown to regulate cell proliferation and migration and is involved in the control of the Hippo pathway [43, 44]. In particular, the Hippo pathway of CSCs plays a significant role in tumorigenesis, chemoresistance, metastatic potential, and self-renewal by promoting CSC characteristics [45, 46]. However, the CSC-specific regulatory mechanisms of the Hippo pathway and AMOTL2 [45] remain unclear, and signalling through CSC-secreted EVs has not been elucidated. Notably, we demonstrated that miR-135b and miR-135a targeted AMOTL2 and the expression of AMOTL2 can be increased through miR-135b and miR-135a inhibition. This result might be a clue that miR-135b and miR-135a of BTSCs-EVs may be able to regulate the Hippo pathway via AMOTL2, a major molecule of the Hippo pathway.

In addition, we observed that high expression of AMOTL2 is associated with prolonged survival in two paediatric medulloblastoma cohorts, particularly in the patients of Group 3 and Group 4. The expression of AMOTL2 may be implicated in the prognosis of certain subgroups. This result also suggests a possible role of AMOTL2 as a tumour suppressor in medulloblastoma Groups 3 and 4.

The limitation of our study is the small number of specimens, which not only is based on a relatively limited incidence of paediatric medulloblastoma but also reflects the difficulty in simultaneously isolating spheroid-forming cells and bulk tumour cells for acquiring a sufficient amount of EVs. Future research is necessary to extend the significance of our findings and to more deeply explore the specific contributions of each miRNA identified here. If the regulation of miR-135b and miR-135a affects the stemness properties of BTSCs, in vivo experiments are required to validate the relevance between the regulation of these miRNAs and tumour progression.

## Conclusion

The present study described the miRNA expression pattern in medulloblastoma BTSCs and EVs derived from BTSCs, as well as their potential role in affecting recipient cells. The study provides new insights into the epigenetic regulation of CSCs in medulloblastoma. These findings may act as a basis for further studies into the discovery of better prognostic markers and the development of promising therapeutics, as well as for deciphering the specific role of EVs in CSC maintenance, regulation and progression.

## Supplementary information

** Additional file 1: Fig. S1. **Apoptosis and senescence analysis by ani-miR-135b and anti-miR-135a treatment in brain tumour spheroid-forming cells (BTSCs). (A) Flow cytometric analysis is applied to determine the ratio of apoptosis with Annexin V-FITC/PI staining. The anti-miR-135b and anti-miR-135a treatment induce the early apoptosis cells compared with anti-miR-NC. (B) Representative images of SA-β-gal staining after ani-miR-135b and anti-miR-135a treatment display no senescence induced cells.

** Additional file 2: Fig. S2. **The correlation coefficients between target genes AMOTL2 and (A) miR-135b and (B) miR-135a in medulloblastoma subgroups of WNT, SHH and Group 3. (C) The R values of STAT6 and GPX8, which are targeted only to miR-135a, were − 0.85386 (P < 0.0001) and − 0.81841 (P < 0.0001), respectively.

** Additional file 3: Fig. S3. **Visualization of AMOTL2 expression pattern in the Hippo signalling pathway and in the tight junction pathway in brain tumour spheroid-forming cells (BTSCs).

## Data Availability

The datasets generated and analyzed during the current study are available from the corresponding author upon reasonable request.
