# Supplementary figures and images for: Extracellular vesicle-associated miR-135b and -135a regulate stemness in Group 4 medulloblastoma cells by targeting angiomotin-like 2
Source: Cancer Cell Int. 2020 Nov 20;20:558. doi: 10.1186/s12935-020-01645-6 (PMC7678136; doi:10.1186/s12935-020-01645-6)

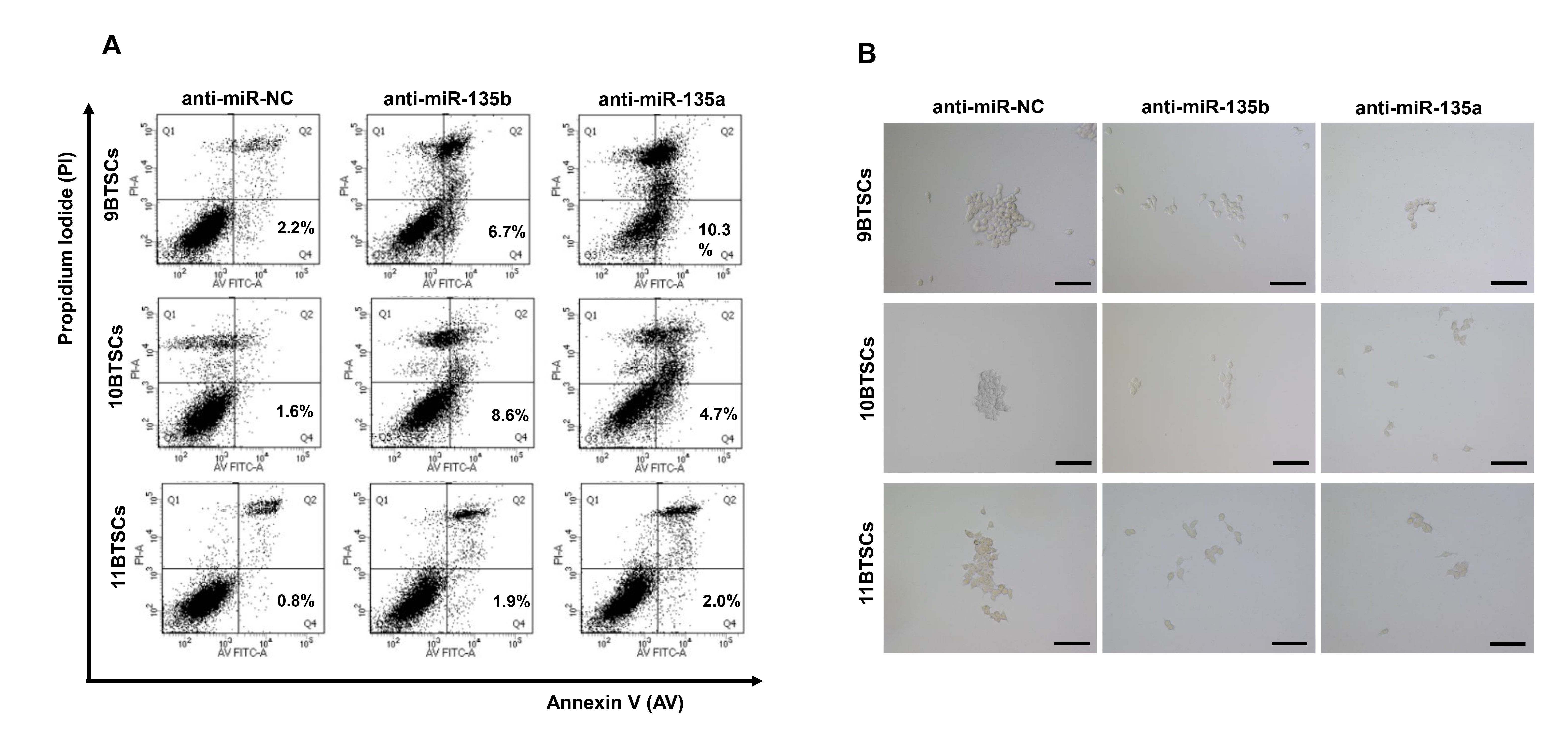

Supplement: Supplementary file 1 — Additional file 1: Fig. S1. Apoptosis and senescence analysis by ani-miR-135b and anti-miR-135a treatment in brain tumour spheroid-forming cells (BTSCs). (A) Flow cytometric analysis is applied to determine the ratio of apoptosis with Annexin V-FITC/PI staining. The anti-miR-135b and anti-miR-135a treatment induce the early apoptosis cells compared with anti-miR-NC. (B) Representative images of SA-β-gal staining after ani-miR-135b and anti-miR-135a treatment display no senescence induced cells. [file 12935_2020_1645_MOESM1_ESM.tif]

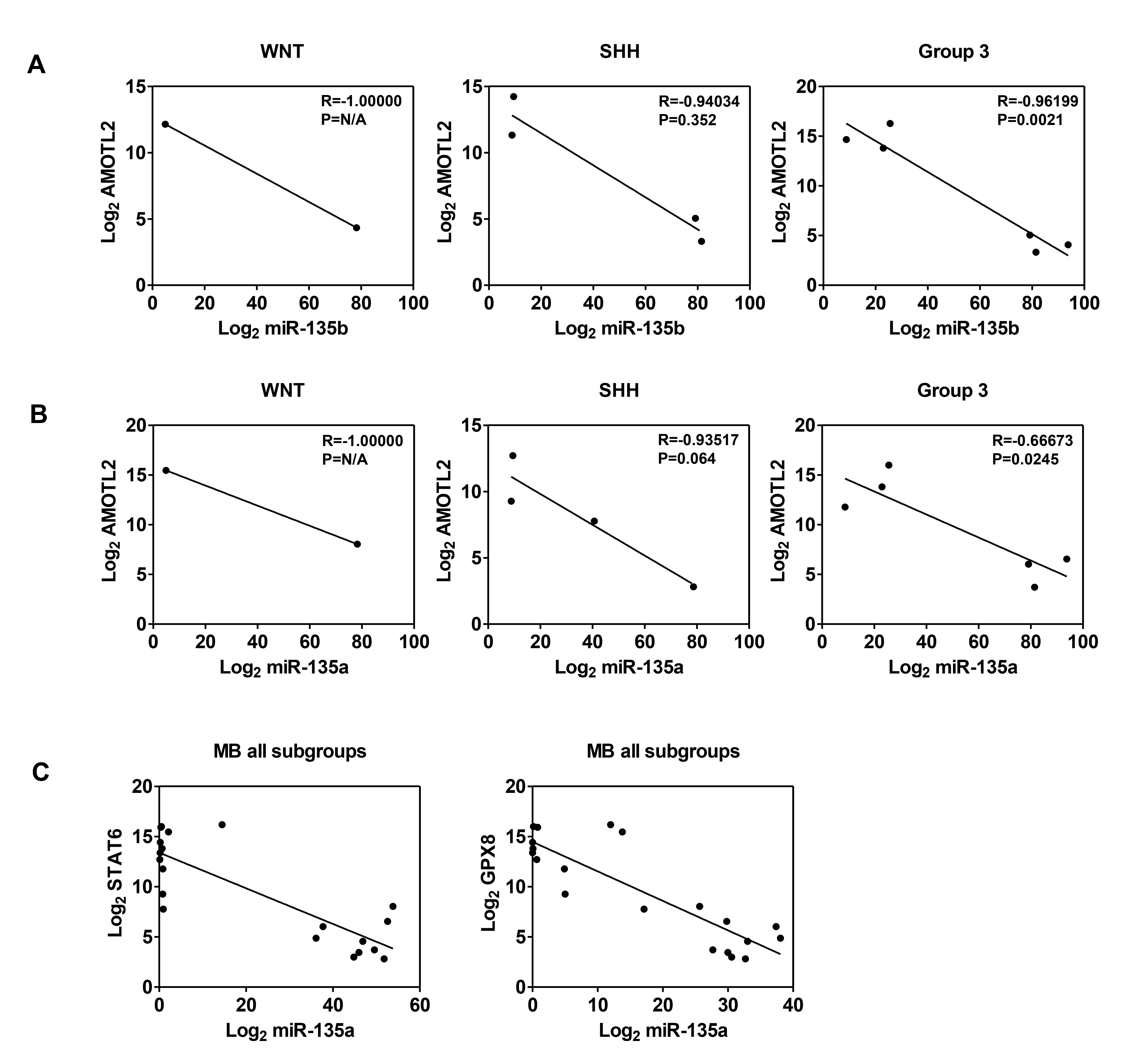

Supplement: Supplementary file 2 — Additional file 2: Fig. S2. The correlation coefficients between target genes AMOTL2 and (A) miR-135b and (B) miR-135a in medulloblastoma subgroups of WNT, SHH and Group 3. (C) The R values of STAT6 and GPX8, which are targeted only to miR-135a, were − 0.85386 (P < 0.0001) and − 0.81841 (P < 0.0001), respectively. [file 12935_2020_1645_MOESM2_ESM.tif]

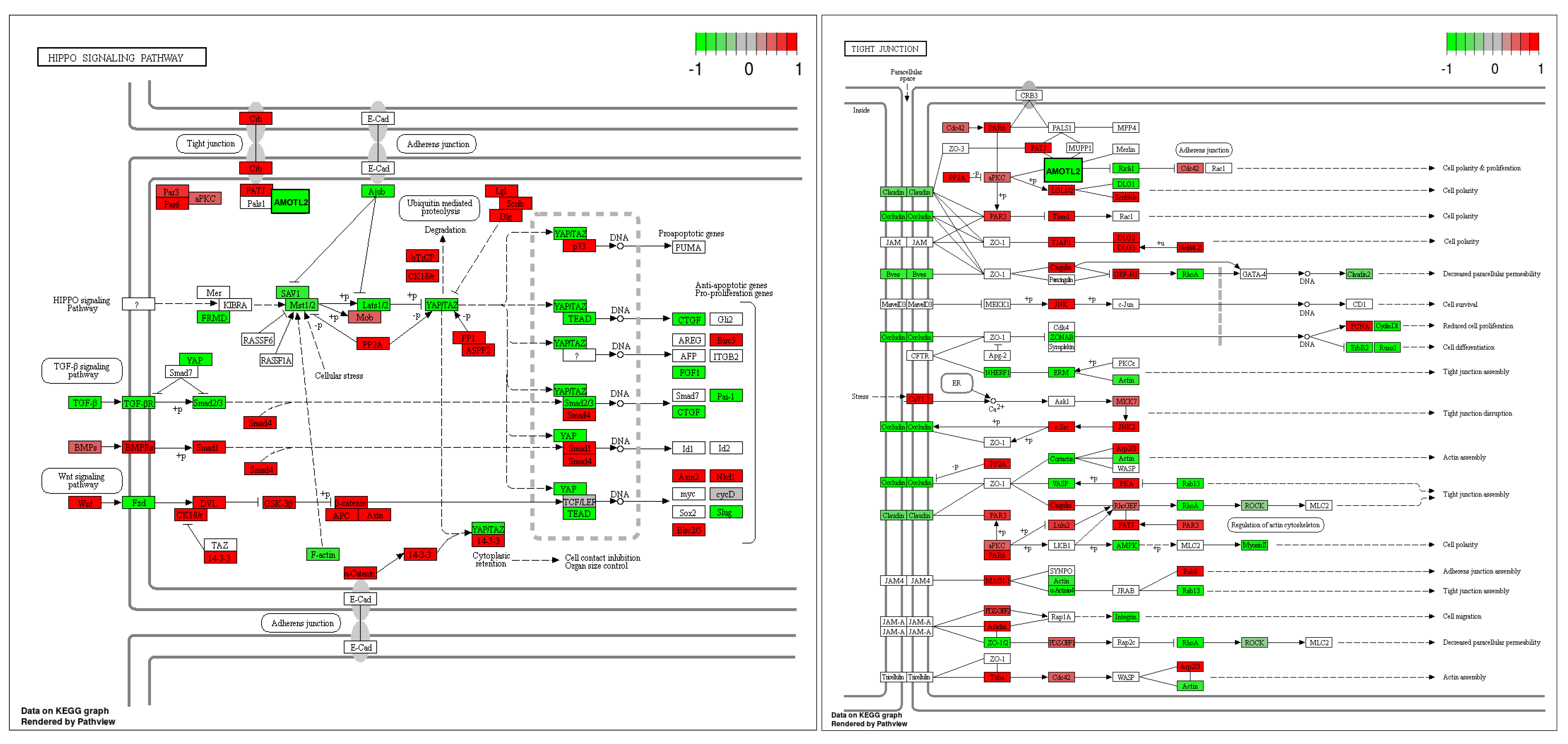

Supplement: Supplementary file 3 — Additional file 3: Fig. S3. Visualization of AMOTL2 expression pattern in the Hippo signalling pathway and in the tight junction pathway in brain tumour spheroid-forming cells (BTSCs). [file 12935_2020_1645_MOESM3_ESM.tif]
